# Supplementary material for: Whole-genome sequencing of 128 camels across Asia reveals origin and migration of domestic Bactrian camels
Source: Commun Biol. 2020 Jan 7;3:1. doi: 10.1038/s42003-019-0734-6 (PMC6946651; doi:10.1038/s42003-019-0734-6)
Supplement: Supplementary file 1 — Supplementary Information [file 42003_2019_734_MOESM1_ESM.pdf]

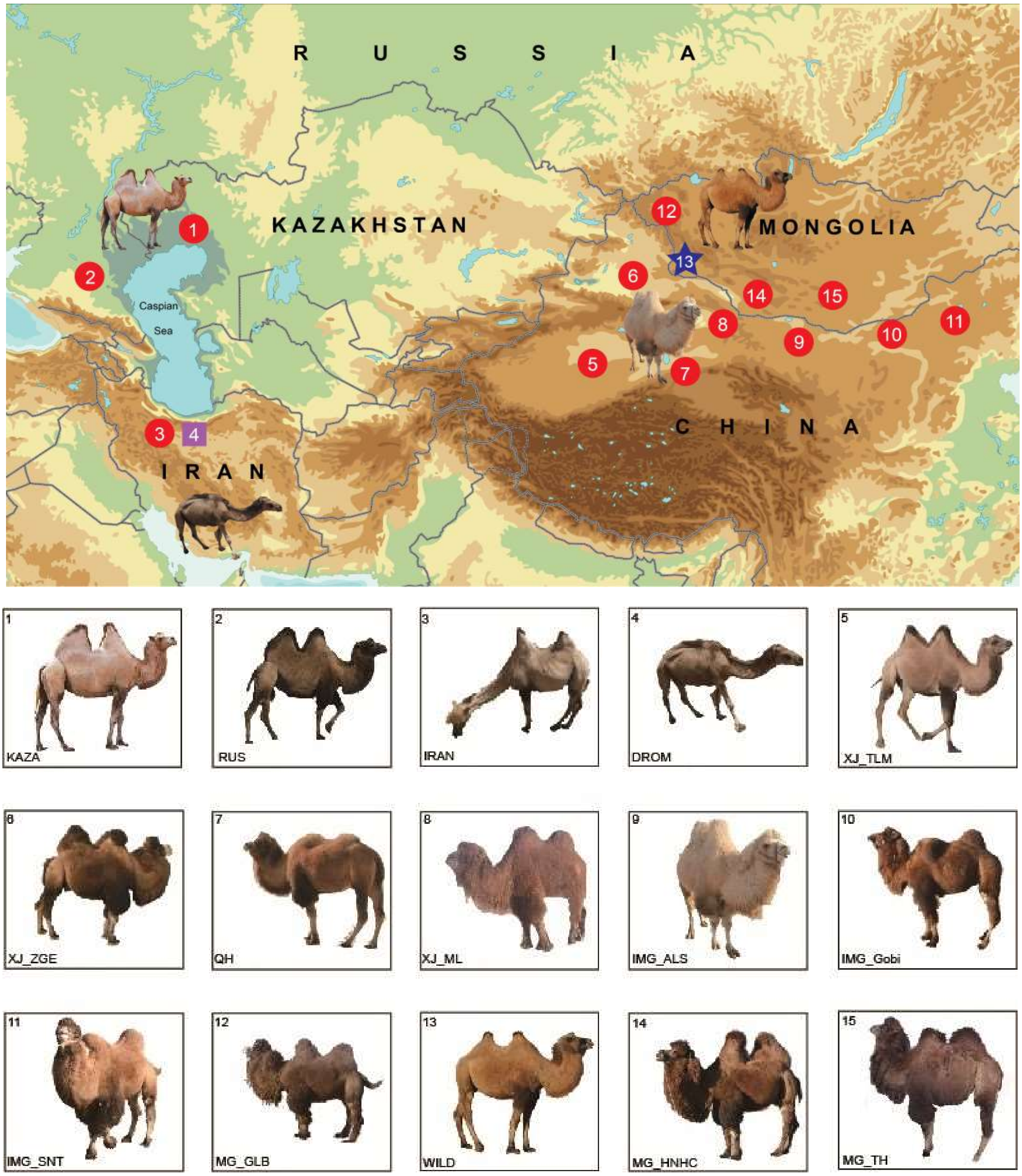

Supplementary Figure 1. Geographic locations of the camel samples. The Bactrian camels are mainly distributed in the Mongolian Plateau (East Asia) and around the Caspian Sea (Central Asia). Populations sampled at each location and their morphological features were shown in the bottom. The population abbreviations and sample size were listed in Supplementary Table 1.

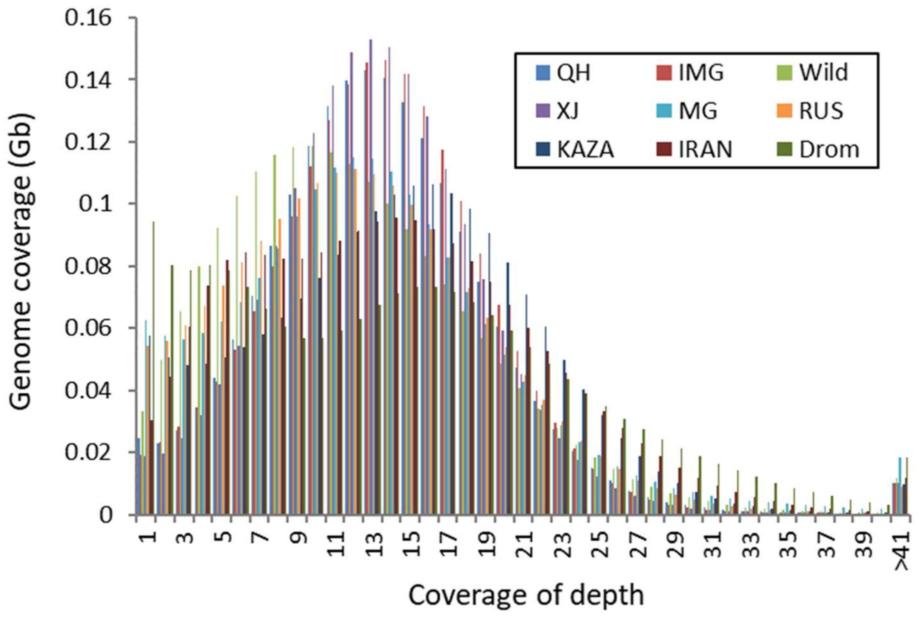

Supplementary Figure 2. Sequencing depth distribution. The sequencing depths of individuals from the same population were averaged for plotting. The mode of the distribution is 13× and most of the bases are covered with a depth ranging from 1 to 40×.

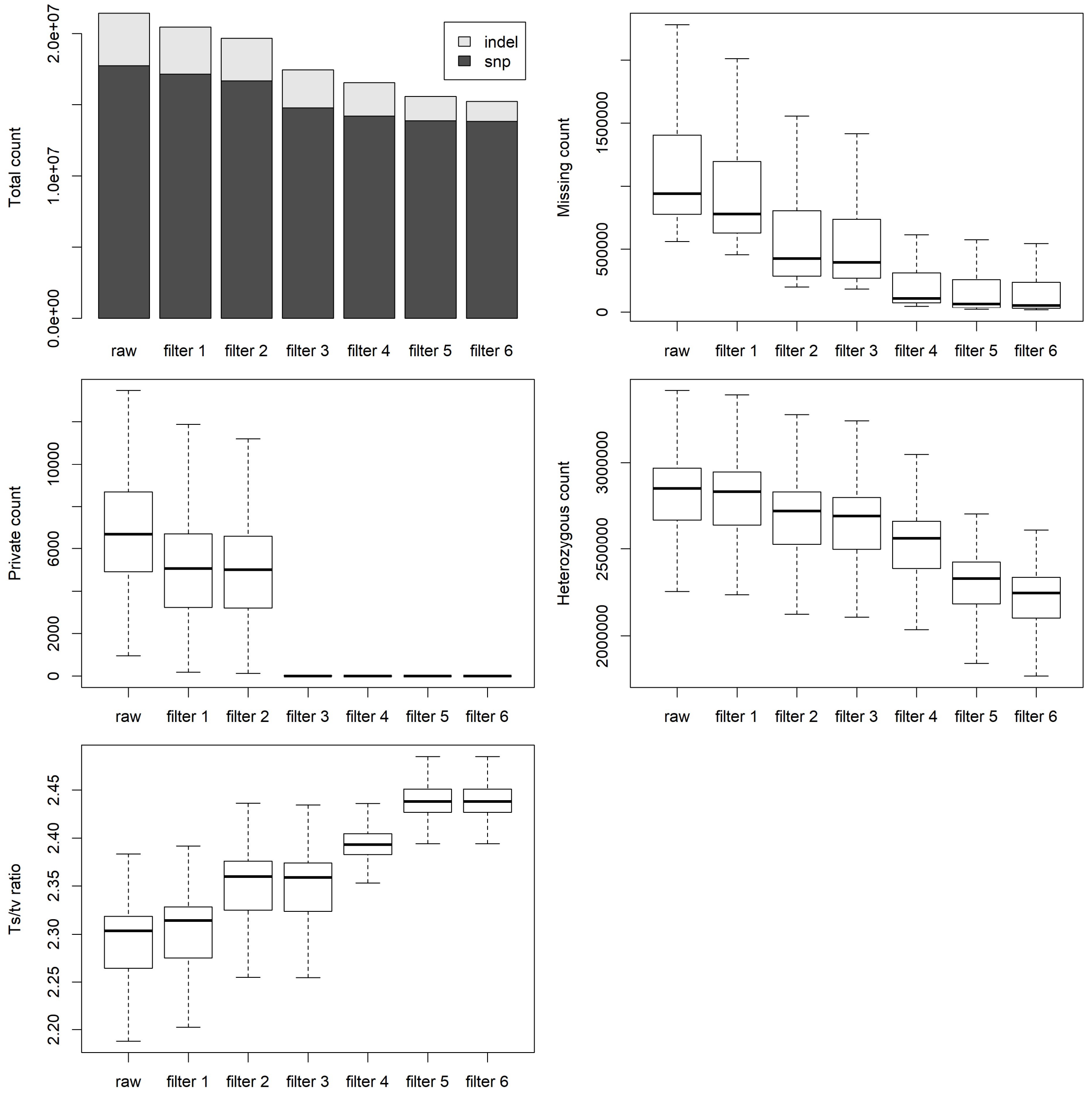

Supplementary Figure 3. Distribution of variant statistics per individual following each filtering step. Ts/tv is the transition to transversion ratio. Filter 1: variant quality score >40; Filter 2: sequencing depth summing all individuals >200 and <5,000; Filter 3: minor allele frequency >1%; Filter 4: variants with <20% individuals with missing genotypes; Filter 5: root mean square of mapping quality >30; Filter 6: biallelic variants.

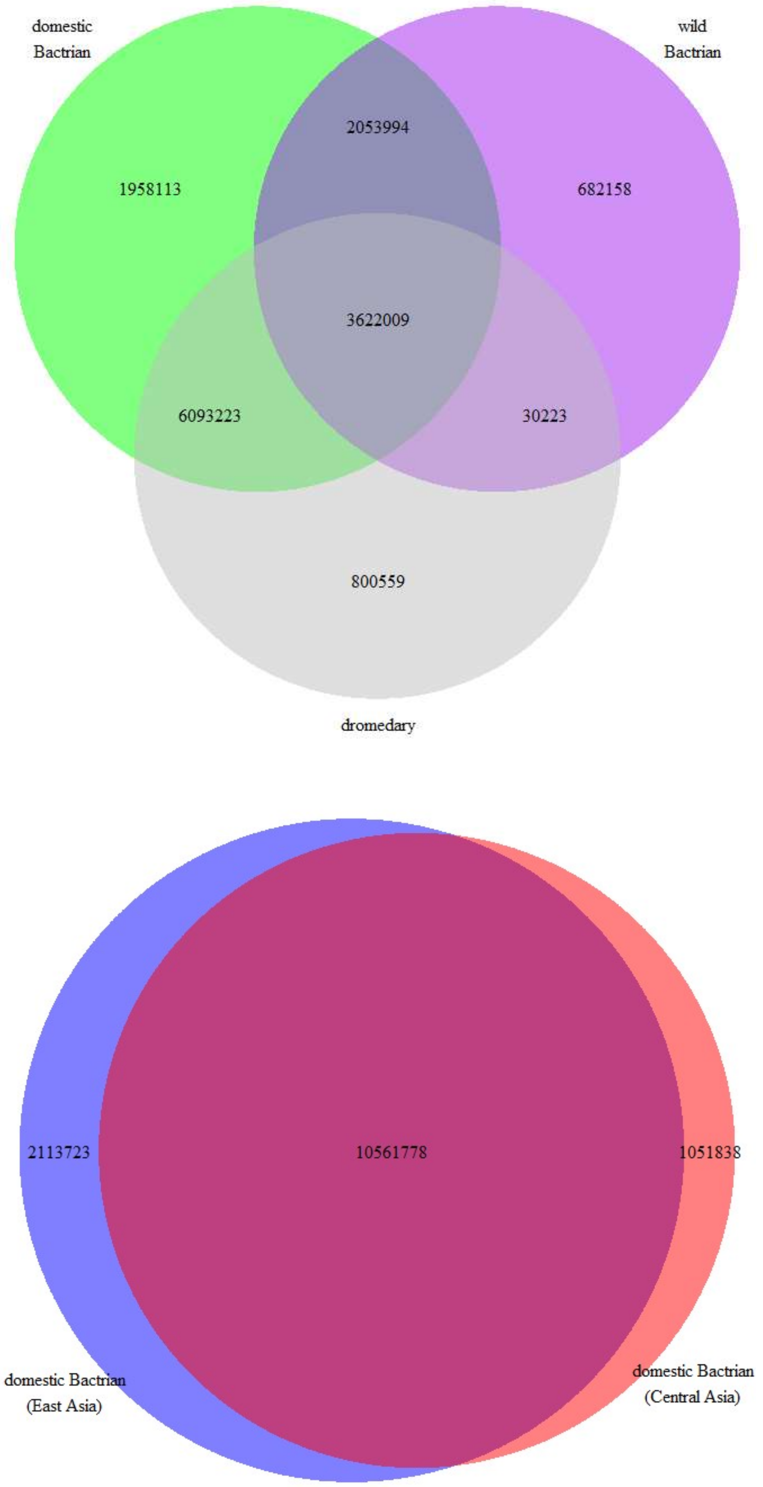

Supplementary Figure 4. Venn diagrams of genomic variants. Upper panel: variants between domestic Bactrian camels, wild Bactrian camels and dromedaries; lower panel: variants between East Asian and Central Asian domestic Bactrian camels.

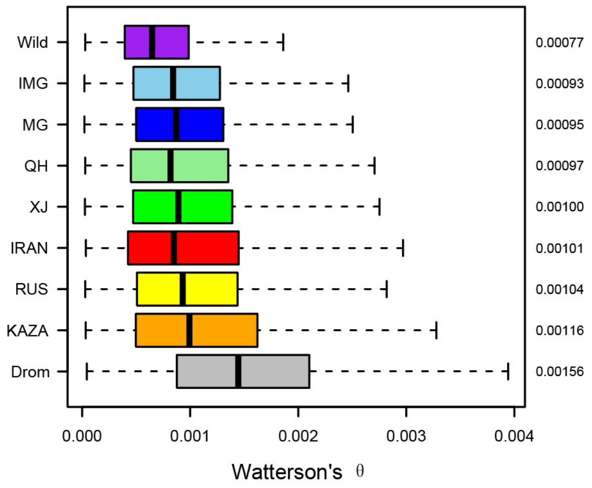

Supplementary Figure 5. Watterson's θ of the camel populations. The statistics were calculated with 10-kb windows across the genome. The means are shown on the right.

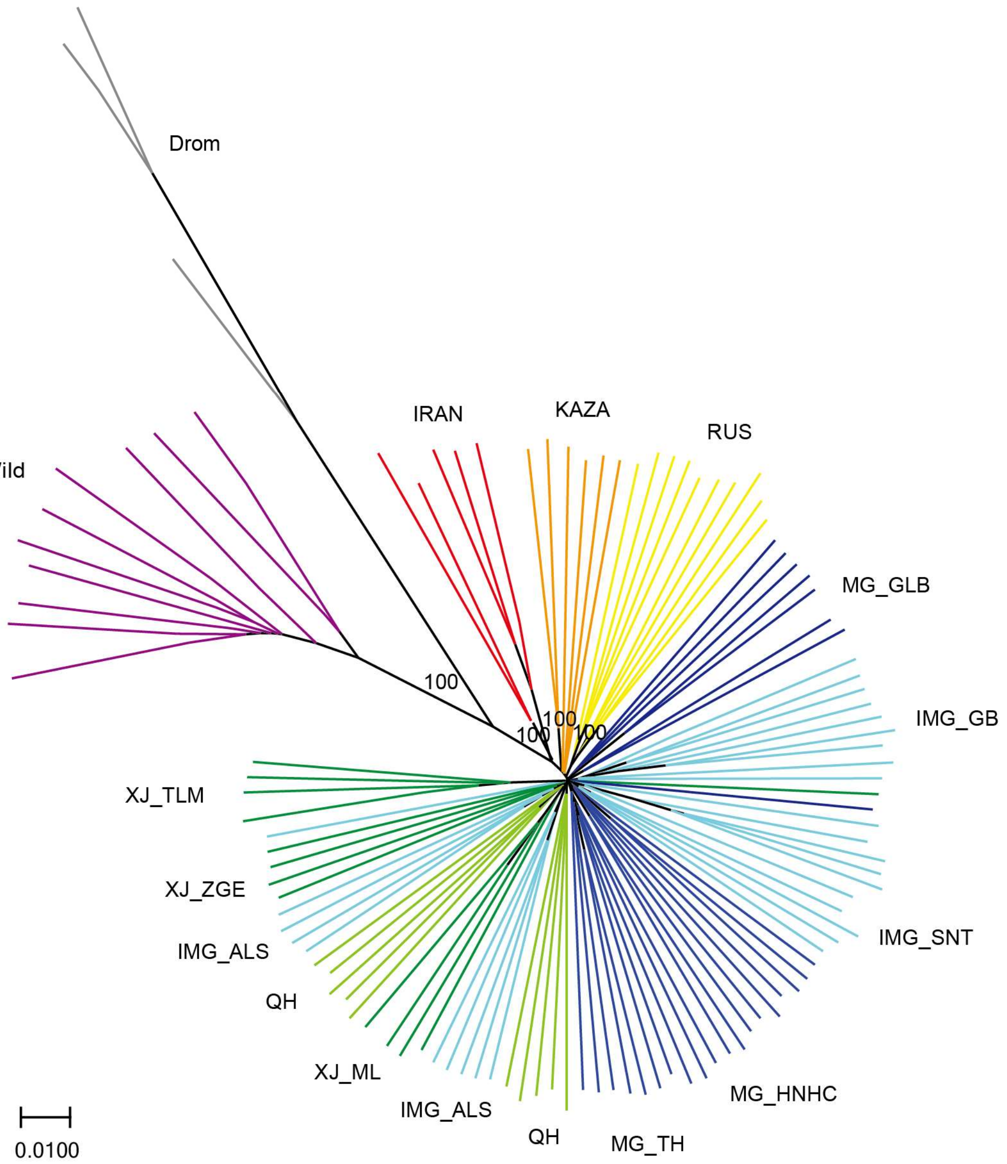

Supplementary Figure 6. Neighbor-joining tree of all camels based on pairwise IBS. Numbers indicate bootstrap values of main branches.

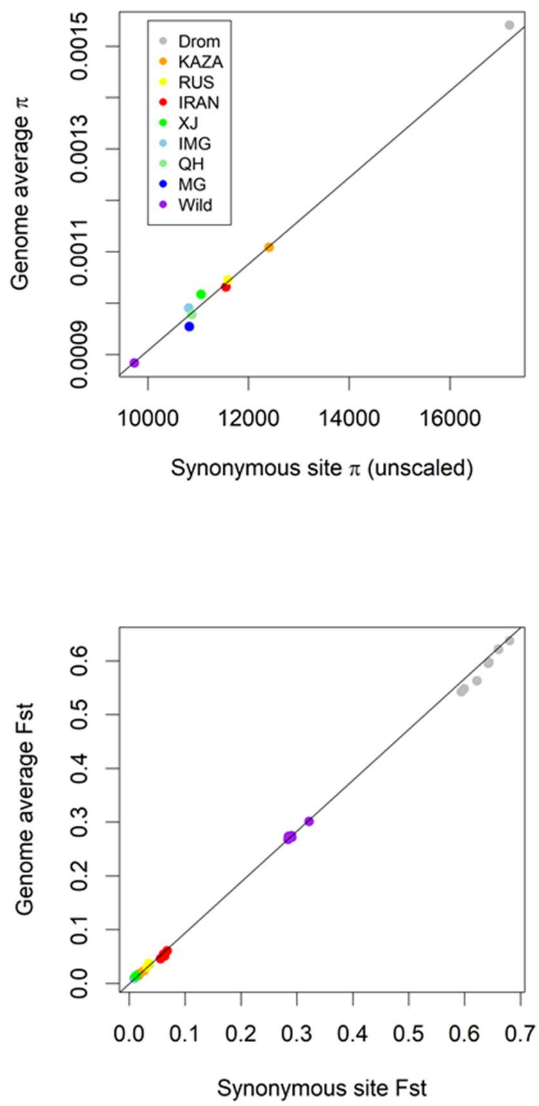

Supplementary Figure 7. Comparison of population genetic statistics between genome-wide SNPs and synonymous SNPs. Upper panel: nucleotide diversity π. The synonymous π is represented by absolute SNP count. Lower panel: pairwise differentiation Fst.

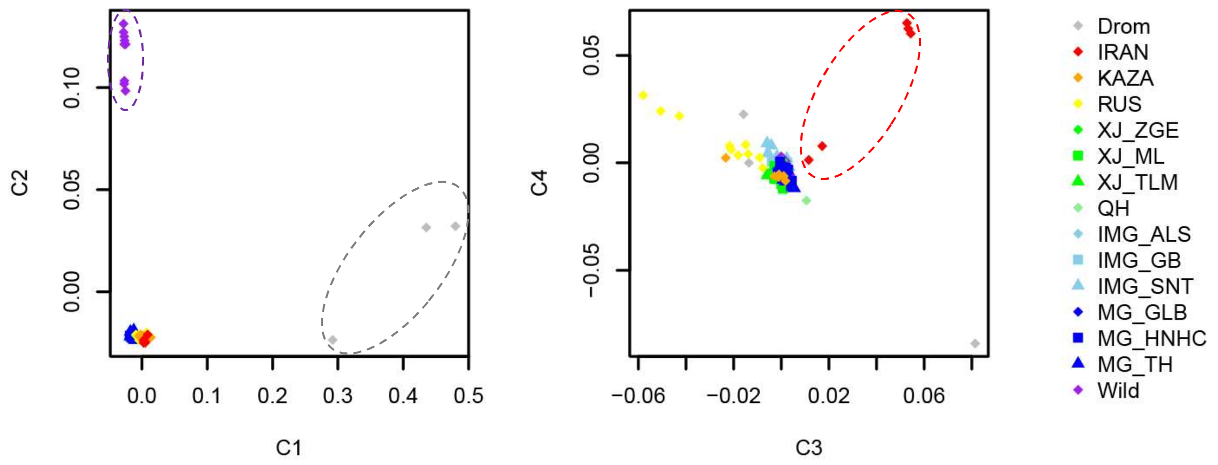

Supplementary Figure 8. Multidimensional scaling (MDS) plot based on all SNPs.

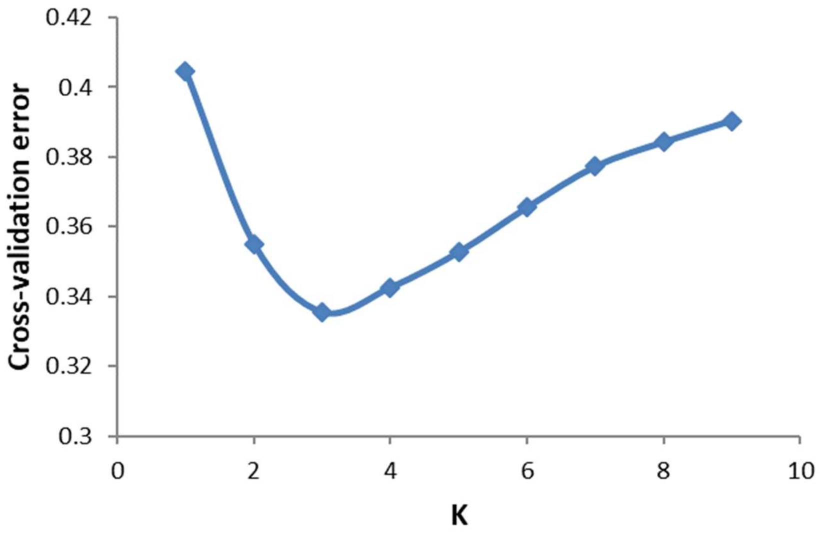

Supplementary Figure 9. Cross-validation errors in the ADMIXTURE analysis. The number of ancestry K was assumed from 1 to 9 and K = 3 is the optimum number.

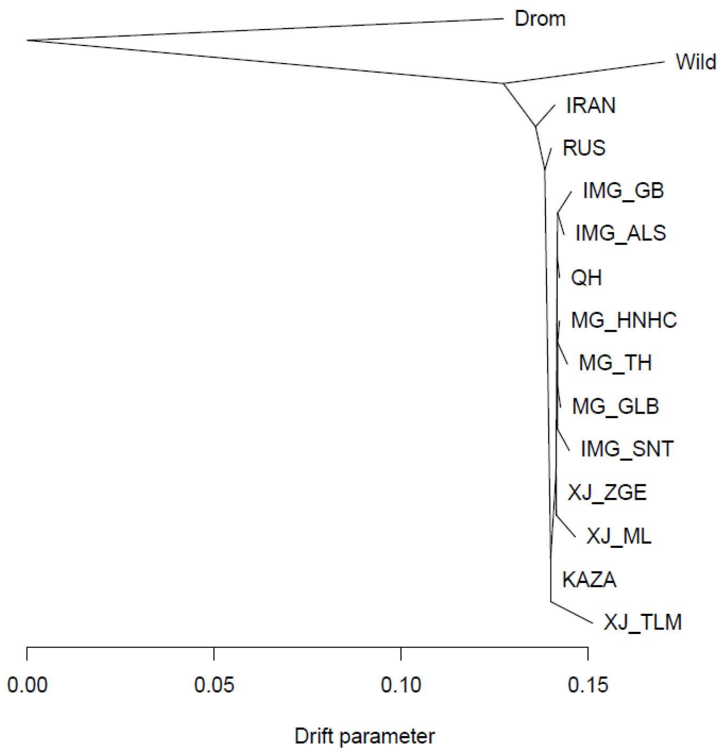

Supplementary Figure 10. Treemix analysis without migration events.

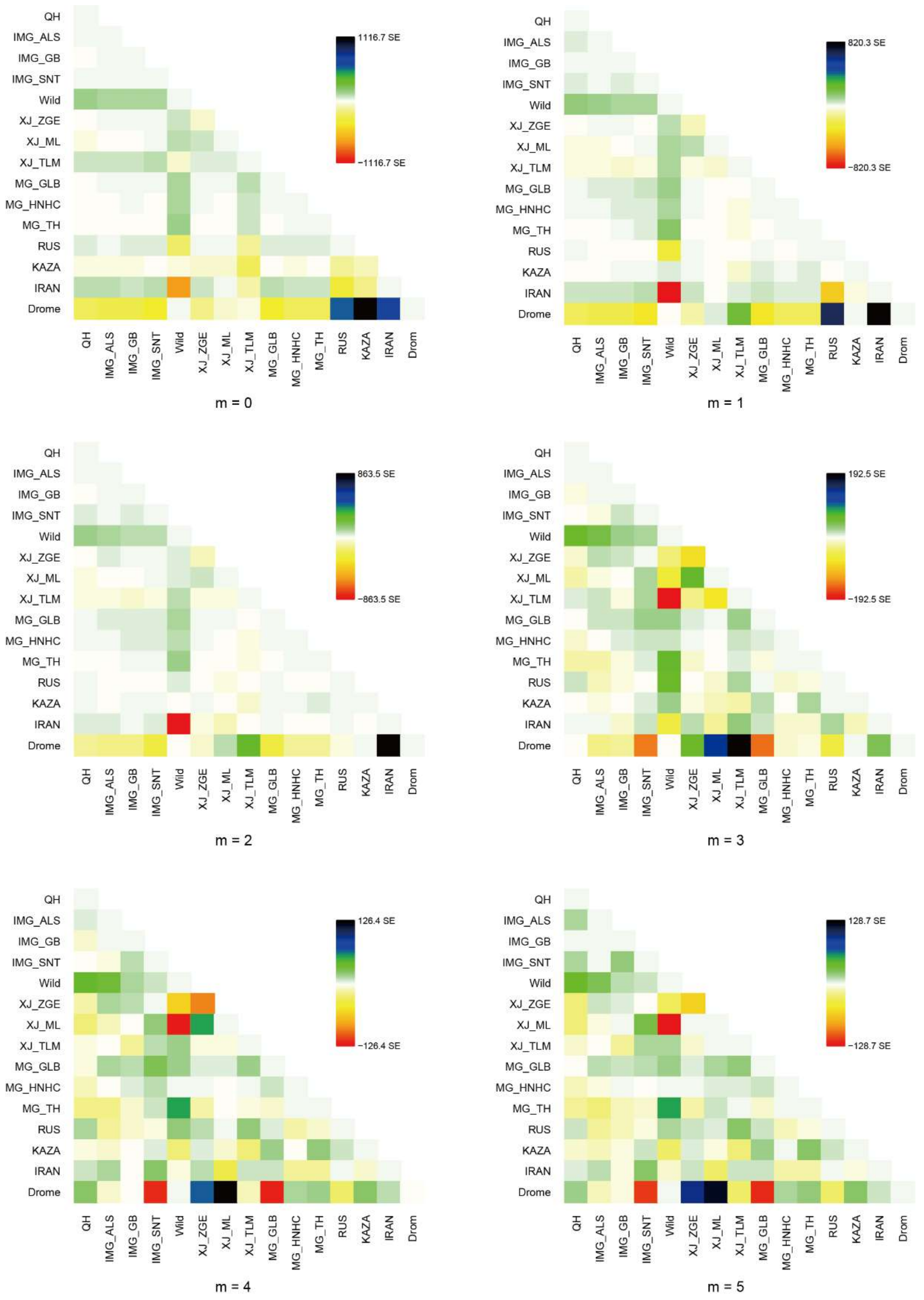

Supplementary Figure 11. Residue visualization for the fit of TreeMix to the data with different number of migration events m. Large positive residues indicate pairs of populations where the fit might be improved by adding additional migration edges.

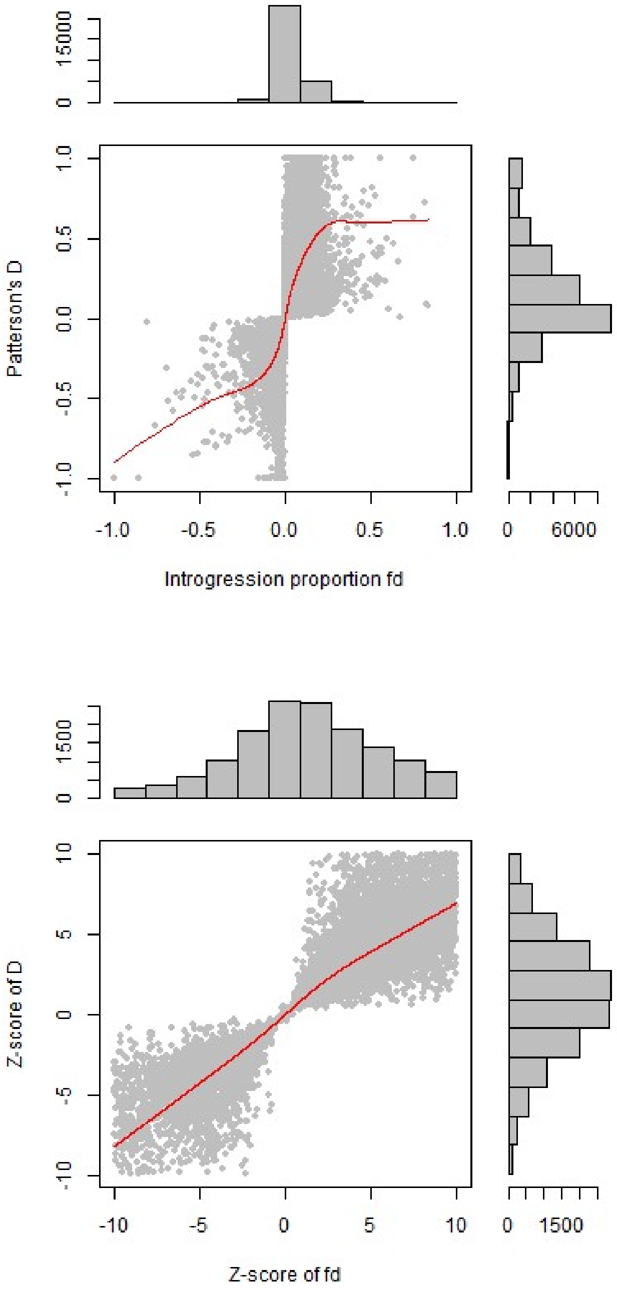

Supplementary Figure 12. Statistics of the BABA/ABBA test. The test was performed for 100-kb sliding windows based on population configuration (East Asian, Central Asian; wild, drom). Upper panel: comparison of Patterson's D and introgression proportion f_d. The latter shows smaller variance than the former. Lower panel: Z-score distribution of the two statistics, which was calculated by the Jackknife procedure with a 10-kb block removed each time.

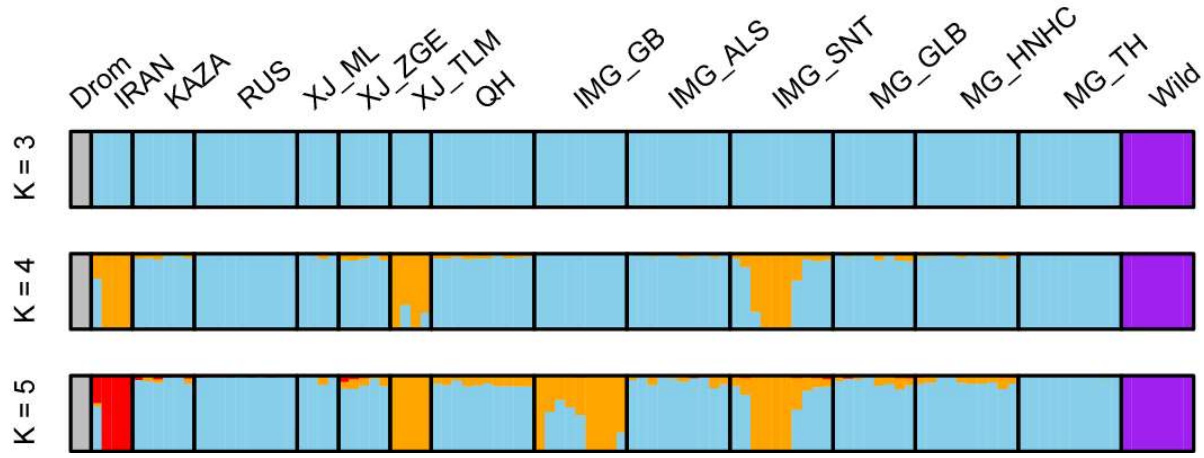

Supplementary Figure 13. Admixture analysis after removing introgression. The ancestry of dromedaries in IRAN/KAZA/RUS was reduced by excluding genomic segments showing Z-score > 2 with the local BABA/ABBA test.

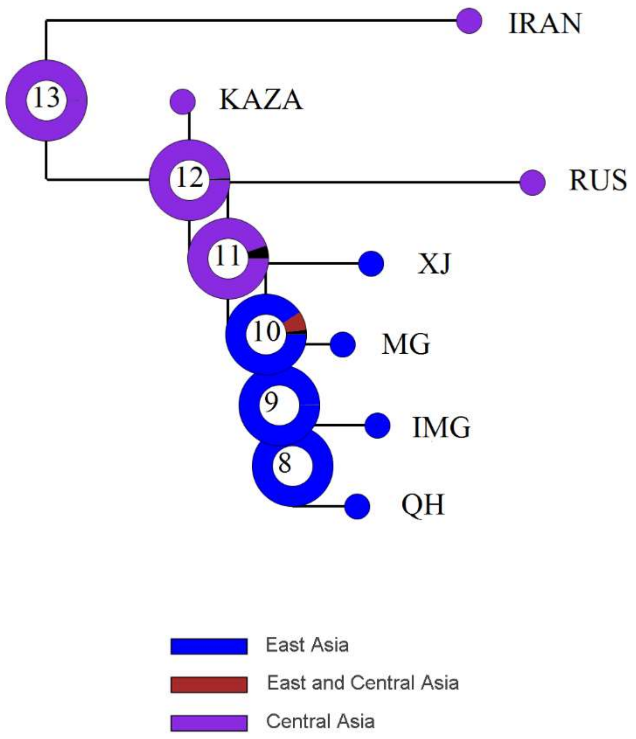

Supplementary Figure 14. Ancestral area inference with the Bayesian binary MCMC analysis.

The probability of an ancestral node assigned to each area is represented by different colors.

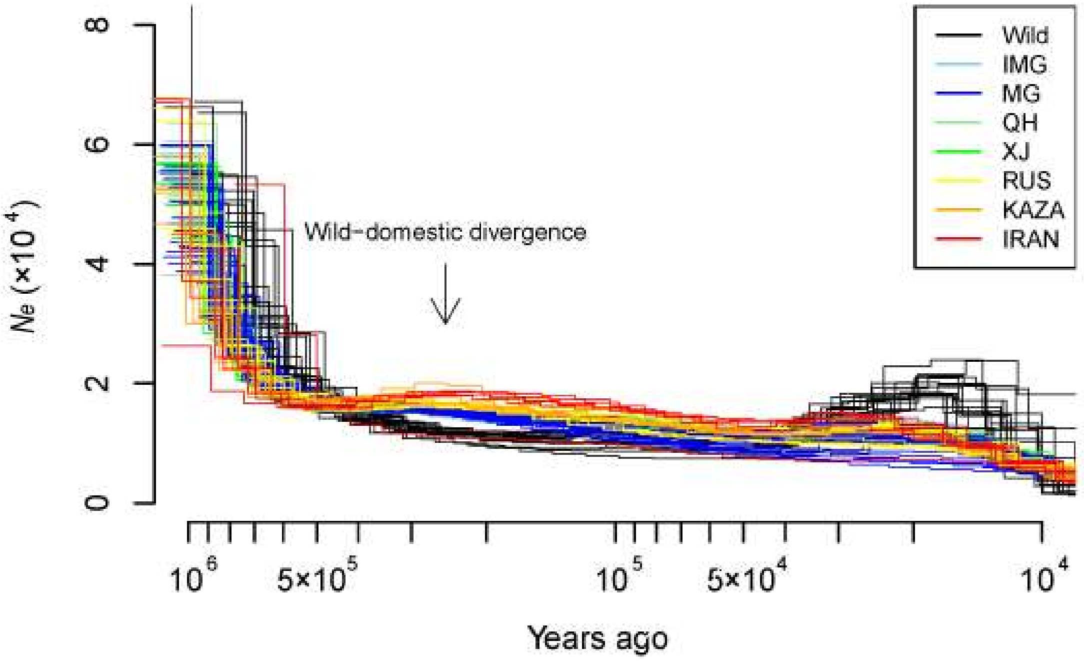

Supplementary Figure 15. Historical effective population size (*N*e) of Bactrian camels inferred by PSMC. Each trajectory is based on one individual genome. The arrow indicates the separation of trajectories between wild and domestic Bactrian camels. To comply with previous studies, the results were scaled with a generation time of 5 years and a mutation rate of 2.5×10^{-8} per site per generation.

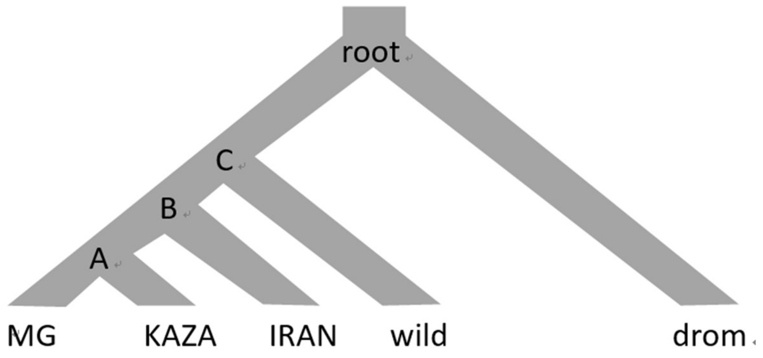

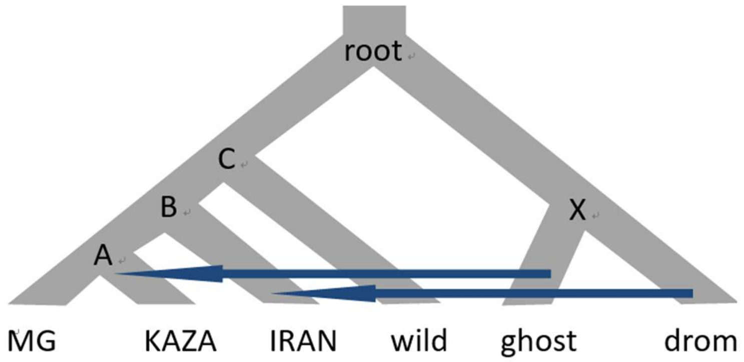

Population	Sample	Sequencing depth
MG	SAMN06759170	15.23
KAZA	SAMN06759203	14.91
IRAN	SAMN06759210	15.56
Wild	SAMN06759127	15.97
Drom	SAMN06759212	16.54

Supplementary Figure 16. Population phylogeny used in the G-PhoCS analysis. Upper panel: Phylogeny without migration based on the Fst distance. Ancestral populations were labeled in the tree. Middle panel: Migration bands from the dromedary to IRAN, and a ghost population related to the dromedary to KAZA. Lower panel: One diploid sample per population was used for G-PhoCS analysis.

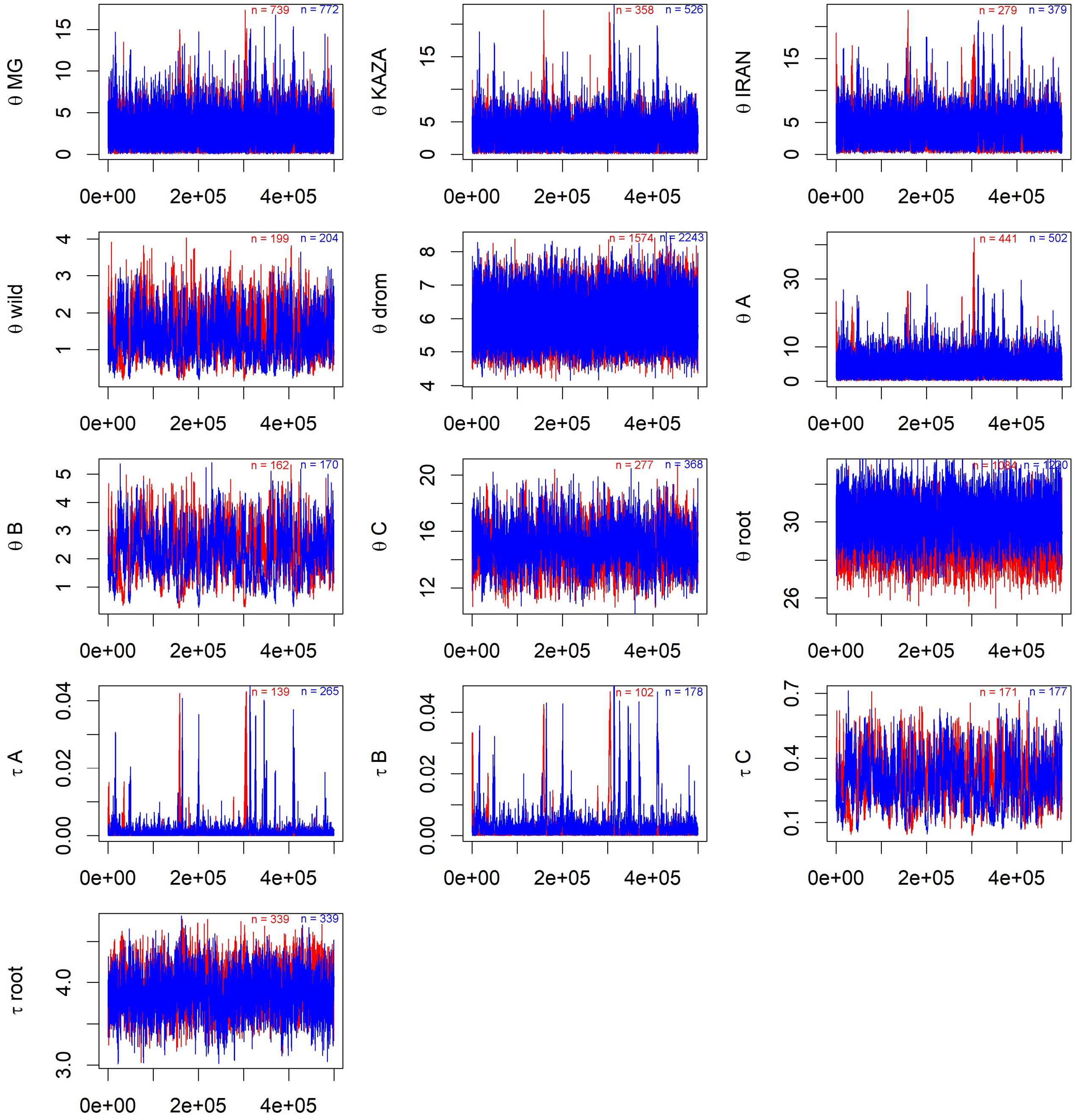

Supplementary Figure 17. MCMC traces in the G-PhoCS analysis without migration. θ and τ are the mutation-scaled effective population size and population divergence time, respectively (scaled up by 10^4). A total of 500,000 iterations with 10 iterations between two traced samples are shown. Two independent runs on independent datasets are represented by two different colors. n is the effective sample size with auto-correlation adjustment by Tracer. The traces were checked for convergence and mixing of MCMC, and the first 1/10 traced samples were discarded.

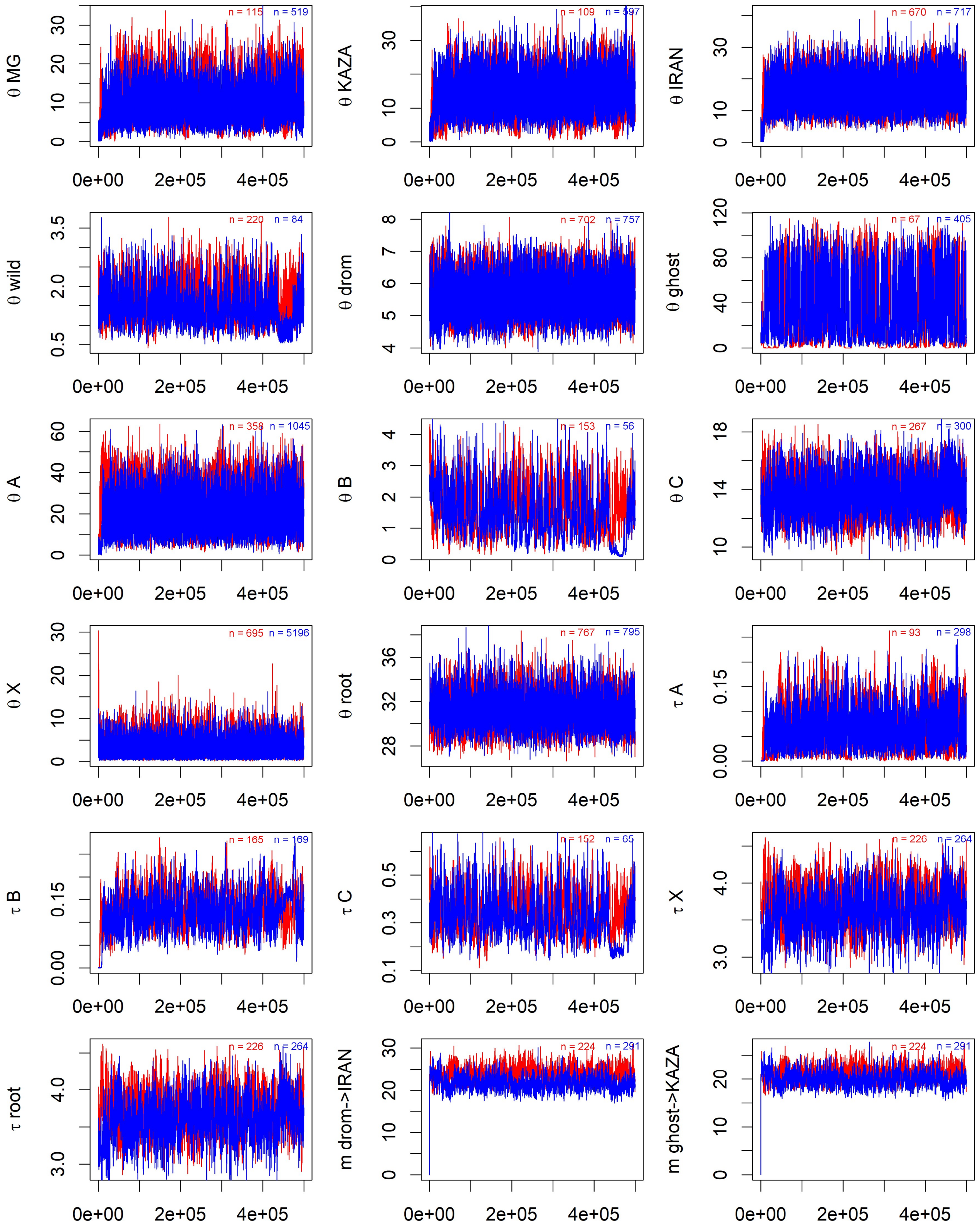

Supplementary Figure 18. MCMC traces in the G-PhoCS analysis with migration. m is the mutation-scaled migration rate per generation (scaled by 0.1). The first 1/10 traced samples were discarded.

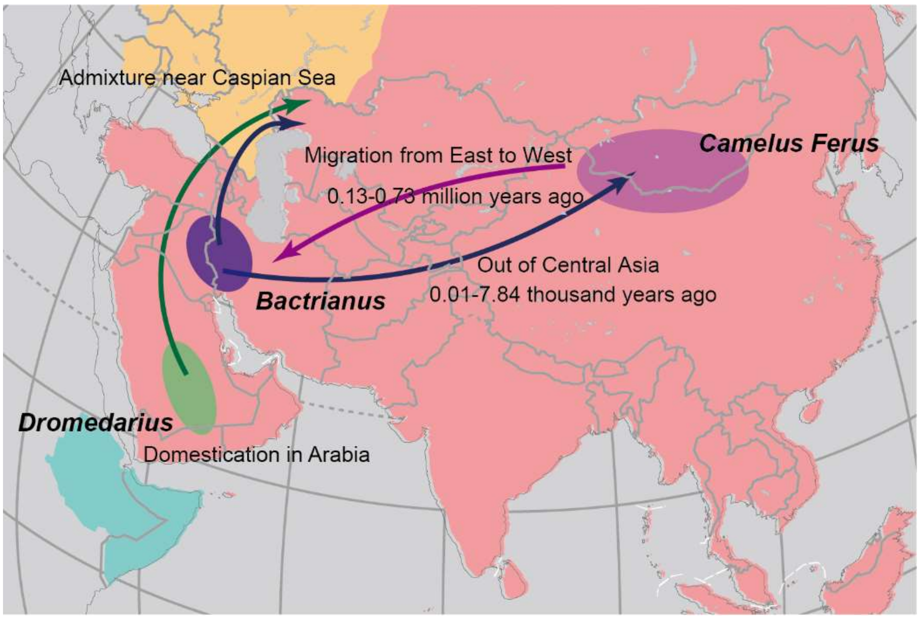

Supplementary Figure 19. A proposed migration route of Bactrian camels across Asia. The timing of the events (represented by 95% confidence interval) were based on the G-PhoCS model without admixture.

Supplementary Table 1.Sample information and summary of sequencing data.

Country	District	Breed (Abbr.)	NCBI sample accession (PRJNA383081)	Sample size	Mean raw bases (Gb)	Mean bases after QC (Gb)
China	Qinghai Province	Qinghai (QH)	SAMN06759087-06759096	10	32.27	32.10
China	Alashan, Inner Mongolia	Alashan (IMG_ALS)	SAMN06759097-06759106	10	33.34	33.24
China	Bayan Nur, Inner Mongolia	Gobi Red (IMG_GB)	SAMN06759107-06759116	10	33.16	33.09
China	Xilingol, Inner Mongolia	Sonid (IMG_SNT)	SAMN06759117-06759126	10	32.85	32.79
Mongolia	Gobi Altai	Wild	SAMN06759127-06759145	19	37.24	33.22
China	Altay Fuyun, XinJiang	Zhungeer (XJ_ZGE)	SAMN06759146-06759150	5	34.91	34.55
China	Mulei Kazakh, XinJiang	Mulei (XJ_ML)	SAMN06759151-06759155	5	31.51	31.43
China	Tarim Basin, XinJiang	Tarim (XJ_TLM)	SAMN06759156-06759160	5	30.60	30.55
Mongolia	Khanbogd and Bayan-ovoo soums, Umnugobi Province	Galbiin Goviin ulaan (MG_GLB)	SAMN06759161-06759168	8	30.99	30.91
Mongolia	Mandal-ovoo soum, Umnugobi Province	Khaniiin khetsiin khuren (MG_HNHC)	SAMN06759169-06759178	10	32.70	32.64
Mongolia	Togrog soum, Gobi-Altai Province	Tokhom-tungalag (MG_TH)	SAMN06759179-06759188	10	31.44	31.38
Russia	The Republic of Tyva, Kalmykia	Kalmyk (RUS)	SAMN06759189-06759198	10	30.79	30.69
Kazakhstan	Almaty Province	Kazakhstan (KAZA)	SAMN06759199-06759204	6	32.40	32.38
Iran	Ardabili	Iran (IRAN)	SAMN06759205-06759210	6	39.19	33.54
Iran	Ardabili	Dromedary	SAMN06759211-06759214	4	41.73	35.60

Supplementary Table 2. Means of genome mapping statistics.

Breed	Mapped reads (M)	Mapped read pairs (M)	Unique read pairs (M)	Properly mapped bases (Gb)	Genome depth	Genome coverage
QH	256.51	126.29	120.52	27.54	13.70	0.96
IMG_ALS	265.42	130.70	124.40	28.51	14.19	0.98
IMG_GB	264.07	130.16	123.36	28.30	14.09	0.98
IMG_SNT	261.68	128.97	122.94	28.32	14.09	0.97
Wild	337.48	161.20	150.09	25.96	12.92	0.98
XJ_ZGE	278.13	137.07	131.26	30.27	15.07	0.98
XJ_ML	250.90	123.52	117.56	26.74	13.31	0.96
XJ_TLM	243.90	120.08	114.99	26.17	13.03	0.97
MG_GLB	246.83	121.25	114.34	25.85	12.86	0.96
MG_HNHC	260.55	128.19	120.21	27.28	13.58	0.94
MG_TH	250.58	123.10	114.81	25.79	12.84	0.93
RUS	244.99	120.60	112.93	25.17	12.53	0.95
KAZA	258.27	127.40	122.79	28.37	14.12	0.94
IRAN	265.42	129.21	104.43	28.02	13.95	0.97
Drom	284.05	137.59	117.32	31.08	15.47	0.94

Supplementary Table 3. Summary of variant annotation.

Variant Type	Genome annotation	Exon annotation	Count
SNP	intergenic		8,741,927
	ncRNA		238,367
	UTR		82,771
	intronic		4,626,590
	splicing		465
	exonic	synonymous	76,746
		nonsynonymous	55,809
		stop altering	659
		unknown	8,051
INDEL	intergenic		874,339
	ncRNA		25,475
	UTR		8,975
	intronic		497,235
	splicing		130
	exonic	frameshift	1,098
		nonframeshift	1,306
		stop altering	43
		unknown	291

Supplementary Table 4. Variant count in domestic populations of Bactrian camels.

Population	Total	Population specific	Average heterozygous
MG	10,447,661	285,412	2,185,229
IMG	10,329,929	300,560	2,153,755
QH	8,452,723	122,367	2,238,246
XJ	9,359,473	218,020	2,272,012
RUS	9,050,851	308,890	2,327,141
KAZA	8,648,950	279,301	2,546,593
IRAN	7,602,512	162,448	2,343,079

Supplementary Table 5. Samples with close relationship inferred by KING.

Breed	Removed sample	Preserved sample	Proportion of zero IBS	Kinship coefficient
Wild	SAMN06759128	SAMN06759129	0.0008	0.2375
Wild	SAMN06759128	SAMN06759134	0.0033	0.195
Wild	SAMN06759128	SAMN06759137	0.0103	0.1817
Wild	SAMN06759128	SAMN06759144	0.0021	0.2777
Wild	SAMN06759128	SAMN06759145	0.0034	0.1961
Wild	SAMN06759129	SAMN06759130	0.0013	0.2561
Wild	SAMN06759136	SAMN06759137	0.0027	0.2514
Wild	SAMN06759136	SAMN06759143	0.01	0.1781
Wild	SAMN06759136	SAMN06759127	0.0022	0.2645
Wild	SAMN06759137	SAMN06759131	0.0032	0.2402
Wild	SAMN06759140	SAMN06759133	0.0018	0.2536
Wild	SAMN06759140	SAMN06759134	0.0027	0.2324
Wild	SAMN06759140	SAMN06759145	0.0027	0.2319
Wild	SAMN06759141	SAMN06759132	0.0033	0.2315
Wild	SAMN06759142	SAMN06759135	0.0064	0.2429
Wild	SAMN06759142	SAMN06759131	0.0087	0.1981
Wild	SAMN06759143	SAMN06759127	0.002	0.2459
Wild	SAMN06759143	SAMN06759139	0.0012	0.2569
Wild	SAMN06759145	SAMN06759134	0.0006	0.428
IMG_GB	SAMN06759107	SAMN06759108	0.0028	0.2417
XJ_ML	SAMN06759153	SAMN06759152	0.0089	0.1783
XJ_TLM	SAMN06759159	SAMN06759156	0.001	0.2584
XJ_TLM	SAMN06759159	SAMN06759158	0.001	0.2408
IRAN	SAMN06759209	SAMN06759207	0.0007	0.2323
Drom	SAMN06759214	SAMN06759212	0.0002	0.3205
Drom	SAMN06759214	SAMN06759213	0.0119	0.1775

Supplementary Table 6. P-values of pairwise t-test for nucleotide diversity.

	KAZA	RUS	IRAN	XJ	IMG	QH	MG	Wild
Drom	0 (44.87) [a]	0 (54.09)	0 (54.56)	0 (58.51)	0 (62.40)	0 (62.89)	0 (66.13)	0 (76.24)
KAZA	-	4.42E-15 (7.85)	7.71E-20 (9.12)	3.78E-32 (11.81)	3.48E-50 (14.92)	7.31E-59 (16.20)	1.15E-78 (18.82)	5.08E-179 (28.68)
RUS	-	-	1.40E-1 (1.48)	5.63E-5 (4.03)	1.19E-12 (7.11)	9.09E-18 (8.59)	4.68E-29 (11.20)	3.07E-101 (21.42)
IRAN	-	-	-	1.43E-2 (2.45)	5.70E-8 (5.43)	4.83E-12 (6.91)	4.23E-21 (9.43)	1.70E-83 (19.41)
XJ	-	-	-	-	2.39E-3 (3.04)	3.87E-6 (4.62)	6.34E-13 (7.20)	1.39E-68 (17.54)
IMG	-	-	-	-	-	9.26E-2 (1.68)	2.00E-5 (4.27)	1.30E-49 (14.83)
QH	-	-	-	-	-	-	1.25E-2 (2.50)	1.91E-37 (12.80)
MG	-	-	-	-	-	-	-	1.14E-25 (10.48)

[a] t-statistics in bracket. The statistics were summarized on 20,000 10-kb windows separated by 100 kb with each other.

Supplementary Table 7. Analysis of variance (ANOVA) of missing count.

	Estimate	Std. error	t value	P value
Dromedary	474854	163575	2.903	0.004376
Wild Bactrian	-54302	79460	-0.683	0.495638
Sequencing depth	-46349	16088	-2.881	0.004673

Supplementary Table 8. Migration weight estimated under different assumption of migration events.

Migration events	Drom→KAZA	Drom→RUS	Drom→IRAN	Drom→XJ_TLM	XJ_ML→XJ_ZGE
m=1	0.0446528				
m=2	0.0924439	0.078125			
m=3	0.0563029	0.0491671	0.0654007		
m=4	0.0745705	0.0648239	0.0398977	0.00940315	
m=5	0.0751376	0.0653303	0.039985	0.00971638	0.104136

Supplementary Table 9. Significant admixture (Z-score ≤ -2) by F3 tests.

Population Configuration	F3 Score	SD	Z-score
IRAN;drom,QH	-0.00088105	0.000171907	-5.12512
IRAN;IMG,drom	-0.00123877	0.000161467	-7.67201
IRAN;MG,drom	-0.00102518	0.000162572	-6.30602
KAZA;drom,QH	-0.00777557	0.000150292	-51.7363
KAZA;drom,RUS	-0.00077399	0.000182596	-4.23883
KAZA;IMG,drom	-0.00813264	0.000138629	-58.6647
KAZA;MG,drom	-0.00810483	0.000133427	-60.7435
KAZA;XJ,drom	-0.00631649	0.000155495	-40.6219
RUS;drom,QH	-0.00332145	0.000145829	-22.7763
RUS;IMG,drom	-0.00354733	0.000135719	-26.1373
RUS;MG,drom	-0.00346461	0.000136317	-25.4158
RUS;XJ,drom	-0.0018768	0.000147383	-12.7342

Supplementary Table 10. Significant admixture (|Z-score| ≥ 2) by F4 tests.

Population Configuration [a]	F4 Score	SD	Z-score
IMG,KAZA;drom,wild	-0.00868	0.000153	-56.8168
IMG,RUS;drom,wild	-0.00712	0.000151	-47.2783
IRAN,IMG;drom,wild	0.010684	0.000213	50.2277
IRAN,KAZA;drom,wild	0.002008	0.00025	8.03812
IRAN,QH;drom,wild	0.010466	0.000221	47.3423
IRAN,RUS;drom,wild	0.003562	0.000245	14.5213
KAZA,QH;drom,wild	0.008458	0.000163	51.8413
KAZA,RUS;drom,wild	0.001554	0.000188	8.24619
MG,IRAN;drom,wild	-0.01062	0.000216	-49.1385
MG,KAZA;drom,wild	-0.00861	0.000147	-58.4852
MG,RUS;drom,wild	-0.00706	0.000152	-46.3373
Drom,wild;RUS,QH	0.006905	0.000158	43.8209
XJ,IMG;drom,wild	0.001755	0.000112	15.7005
XJ,IRAN;drom,wild	-0.00893	0.000226	-39.5317
XJ,KAZA;drom,wild	-0.00692	0.000168	-41.1644
XJ,MG;drom,wild	0.00169	0.000108	15.6731
XJ,QH;drom,wild	0.001538	0.000131	11.7575
XJ,RUS;drom,wild	-0.00537	0.00016	-33.5799

[a] Configuration (Y, Z; wild, drom) is considered.

Supplementary Table 11. Additional full length mtDNA sequences used for reconstructing the phylogenetic tree.

GenBank accession	Species	Sequence length (bp)	Origin
NC_009628	Camelus bactrianus	16659	Inner Mongolia, China
KX554925	Camelus bactrianus	16659	Iran
KX554926	Camelus bactrianus	16856	Iran
KX554927	Camelus bactrianus	16669	Iran
KX554928	Camelus bactrianus	16659	Iran
KX554929	Camelus bactrianus	16659	Iran
KX554930	Camelus bactrianus	16659	Iran
AP003423	Camelus bactrianus	16663	Unknown
EF507798	Camelus bactrianus	16659	Umnugobi, Mongolia
EF507799	Camelus bactrianus	16667	Gobi-Altai, Mongolia
EF212037	Camelus bactrianus	16659	Inner Mongolia, China
MH109991	Camelus bactrianus	15432	Iran
MH109997	Camelus bactrianus	15435	Iran
NC_009629	Camelus ferus	16680	Gobi area, Mongolia
EF212038	Camelus ferus	16680	Gobi-Altai, Mongolia
EF507801	Camelus ferus	16651	Gobi-Altai, Mongolia
EF507800	Camelus ferus	16670	Gobi-Altai, Mongolia
NC_009849	Camelus dromedarius	16643	United Arab Emirates: Dubai
KX554931	Camelus dromedarius	16643	Iran
KX554932	Camelus dromedarius	16642	Iran
KX554933	Camelus dromedarius	16622	Iran
KX554934	Camelus dromedarius	16642	Iran
KU605072	Camelus dromedarius	16379	Qatar, Jordan border
KU605073	Camelus dromedarius	16379	Saudi Arabia
KU605074	Camelus dromedarius	16379	Saudi Arabia
KU605075	Camelus dromedarius	16379	Saudi Arabia
KU605076	Camelus dromedarius	16379	Unknown
KU605077	Camelus dromedarius	16379	United Arab Emirates: Dubai
KU605078	Camelus dromedarius	16375	Kenya
KU605079	Camelus dromedarius	16379	Sudan
KU605080	Camelus dromedarius	16379	Pakistan
JN632608	Camelus dromedarius	16665	Morocco

EU159113	Camelus dromedarius	16643	United Arab Emirates: Dubai
MH109998	Camelus dromedarius	15432	Ardabili, Iran
MH109999	Camelus dromedarius	15432	Ardabili, Iran
MH110000	Camelus dromedarius	15431	Ardabili, Iran
MH110003	Camelus dromedarius	15434	Ardabili, Iran
MH110004	Camelus dromedarius	15431	Ardabili, Iran
MH110005	Camelus dromedarius	15432	Ardabili, Iran

Supplementary Table 12. G-PhoCS setup parameters.

Model	Parameter	Value
General settings	num-loci	10000
	burn-in	100000
	mcmc-iterations	500000
	mcmc-sample-skip	10
	locus-mut-rate	CONST
	find-finetunes	TRUE
	find-finetunes-num-steps	100
	find-finetunes-samples-per-step	100
	tau-theta-print	10000
	prior for all θ parameters	$\Gamma(\alpha = 1, \beta = 10000)$
No migration	prior for τ A: (MG, KAZA)	$\Gamma(\alpha = 1, \beta = 100000)$
	prior for τ B: (A, IRAN)	$\Gamma(\alpha = 1, \beta = 100000)$
	prior for τ C: (B, wild)	$\Gamma(\alpha = 1, \beta = 10000)$
	prior for τ root: (C, drom)	$\Gamma(\alpha = 1, \beta = 1000)$
Migration	mig-rate-print	0.1
	prior for all m parameters	$\Gamma(\alpha = 0.002, \beta = 0.00001)$
	migration bands	drom->IRAN, ghost->KAZA
	prior for τ A: (MG, KAZA)	$\Gamma(\alpha = 1, \beta = 100000)$
	prior for τ B: (A, IRAN)	$\Gamma(\alpha = 1, \beta = 100000)$
	prior for τ C: (B, wild)	$\Gamma(\alpha = 1, \beta = 10000)$
	prior for τ X: (drom, ghost)	$\Gamma(\alpha = 1, \beta = 1000)$
	prior for τ root: (C, X)	$\Gamma(\alpha = 1, \beta = 1000)$

Supplementary Table 13. Parameter estimates of G-PhoCS summarized by Tracer.

Parameter [a]	No migration			Migration		
	Mean	Stdev	95% HPD interval [b]	Mean	Stdev	95% HPD interval
θ MG	2.23	1.65	[0.09, 5.41]	9.04	3.85	[2.28, 16.63]
θ KAZA	2.58	1.93	[0.10, 6.31]	13.78	4.89	[4.96, 23.59]
θ IRAN	3.30	2.24	[0.25, 7.61]	15.43	4.49	[7.10, 24.36]
θ wild	1.28	0.49	[0.40, 2.25]	1.40	0.42	[0.66, 2.21]
θ drom	5.75	0.52	[4.78, 6.86]	5.41	0.49	[4.51, 6.44]
θ ghost	-	-	-	31.77	23.95	[0.16, 73.79]
θ A	3.82	3.00	[0.13, 9.21]	18.89	7.99	[4.60, 34.54]
θ B	2.17	0.80	[0.71, 3.74]	1.36	0.74	[9.80E-2, 2.67]
θ C	14.78	1.21	[12.32, 17.07]	13.48	1.15	[11.16, 15.68]
θ X	-	-	-	2.76	1.66	[0.30, 6.05]
θ root	30.19	0.96	[28.31, 32.10]	30.95	1.12	[28.82, 33.16]
τ A	1.61E-3	3.66E-3	[1.00E-5,5.26E-3]	7.90E-2	4.06E-2	[8.32E-3, 0.16]
τ B	2.99E-3	5.56E-3	[5.00E-5,1.18E-2]	0.13	3.90E-2	[5.67E-2, 0.21]
τ C	0.29	0.11	[0.09, 0.49]	0.31	8.80E-2	[0.16, 0.47]
τ X	-	-	-	3.60	0.26	[3.09, 4.10]
τ root	3.85	0.22	[3.41, 4.29]	3.61	0.26	[3.09, 4.11]
m drom->IRAN[c]	-	-	-	21.54	1.57	[18.71, 24.77]
m ghost->KAZA[d]	-	-	-	19.83	1.44	[17.23, 22.81]

[a] θ and τ are scaled by 10^4, m are scaled by 0.1.

[b] Highest posterior density interval. The HPD is a credible set that contains 95% of the sampled values.

[c] Total migration rate for the band $M_{drom\text{->}IRAN} = m_{drom\text{->}IRAN} \times \tau_B$

[d] Total migration rate for the band $M_{ghost\text{->}KAZA} = m_{ghost\text{->}KAZA} \times \tau_A$
