# Supplementary material for: Whole-genome sequencing of 128 camels across Asia reveals origin and migration of domestic Bactrian camels
Source: Commun Biol. 2020 Jan 7;3:1. doi: 10.1038/s42003-019-0734-6 (PMC6946651; doi:10.1038/s42003-019-0734-6)
Supplement: Supplementary file 2 — Reporting Summary [file 42003_2019_734_MOESM2_ESM.pdf]

nature research

Corresponding author(s): Zhen Wang

Last updated by author(s): Dec 4, 2019

Reporting Summary

Nature Research wishes to improve the reproducibility of the work that we publish. This form provides structure for consistency and transparency in reporting. For further information on Nature Research policies, see Authors & Referees and the Editorial Policy Checklist.

Statistics

For all statistical analyses, confirm that the following items are present in the figure legend, table legend, main text, or Methods section.

n/a	Confirmed	
☐	☒	The exact sample size (*n*) for each experimental group/condition, given as a discrete number and unit of measurement
☐	☒	A statement on whether measurements were taken from distinct samples or whether the same sample was measured repeatedly
☐	☒	The statistical test(s) used AND whether they are one- or two-sided<br>*Only common tests should be described solely by name; describe more complex techniques in the Methods section.*
☐	☒	A description of all covariates tested
☒	☐	A description of any assumptions or corrections, such as tests of normality and adjustment for multiple comparisons
☐	☒	A full description of the statistical parameters including central tendency (e.g. means) or other basic estimates (e.g. regression coefficient) AND variation (e.g. standard deviation) or associated estimates of uncertainty (e.g. confidence intervals)
☐	☒	For null hypothesis testing, the test statistic (e.g. *F*, *t*, *r*) with confidence intervals, effect sizes, degrees of freedom and *P* value noted<br>*Give P values as exact values whenever suitable.*
☐	☒	For Bayesian analysis, information on the choice of priors and Markov chain Monte Carlo settings
☒	☐	For hierarchical and complex designs, identification of the appropriate level for tests and full reporting of outcomes
☒	☐	Estimates of effect sizes (e.g. Cohen's *d*, Pearson's *r*), indicating how they were calculated

Our web collection on statistics for biologists contains articles on many of the points above.

Software and code

Policy information about availability of computer code

Data collection	Illumina HiSeq sequencing controlled by HiSeq Control Software.
Data analysis	BWA-MEM (v0.7.12), SAMtools (v1.3.1), GATK (v3.2-2), Picard (v1.135), ANNOVAR (v2013-06-21), VCFtools (v0.1.12b), KING (v2.1.3), PLINK (v1.07), Phylip (v3.69), ADMIXTURE (v1.3.0), TreeMix (v1.12), MEGA (v6.06), RASP (v4.0), G-PhoCS (v1.3), Tracer (v1.6), R(v3.4.2)

For manuscripts utilizing custom algorithms or software that are central to the research but not yet described in published literature, software must be made available to editors/reviewers. We strongly encourage code deposition in a community repository (e.g. GitHub). See the Nature Research guidelines for submitting code & software for further information.

Data

Policy information about availability of data

All manuscripts must include a data availability statement. This statement should provide the following information, where applicable:
- Accession codes, unique identifiers, or web links for publicly available datasets
- A list of figures that have associated raw data
- A description of any restrictions on data availability

The raw data generated from this study have been submitted to the NCBI Sequence Read Archive (http://www.ncbi.nlm.nih.gov/sra/) under accession number SRP107089. The raw data are also available from NODE (http://www.biosino.org/node/) under accession number OEP000024. The datasets to reproduce the main figures have been submitted to Dryad (https://doi.org/10.5061/dryad.tx95x69sz).

Field-specific reporting

Please select the one below that is the best fit for your research. If you are not sure, read the appropriate sections before making your selection.

☐ Life sciences ☐ Behavioural & social sciences ☒ Ecological, evolutionary & environmental sciences

For a reference copy of the document with all sections, see nature.com/documents/nr-reporting-summary-flat.pdf

Ecological, evolutionary & environmental sciences study design

All studies must disclose on these points even when the disclosure is negative.

Study description	We performed whole-genome sequencing of 128 camels across Asia, including representative populations of 105 domestic Bactrian camels, as well as 19 extant wild Bactrian camels and 4 dromedaries.
Research sample	The domestic Bactrian camels included 55 from Inner Mongolia, Xinjiang and Qinghai of China, 28 from Mongolia, 6 from Kazakhstan, 10 from Russia and 6 from Iran. 19 wild Bactrian camels were collected from Gobi-Altai region in Mongolia, and 4 dromedaries were collected from Iran.
Sampling strategy	No sample size calculation was performed, though the domestic Bactrian camels were chosen to cover as many major geographic regions as possible. An endeavor was made to collect samples from unrelated individuals based on the information provided by the owners and local farmers.
Data collection	DNA samples derived from each individual camel were sequenced separetely on Illumina HiSeq by BasePair Biotechnology Co., Ltd.
Timing and spatial scale	The samples were collected during 2010-2015, from the Mongolian Plateau to the Caspian Sea.
Data exclusions	We removed 14 camels showing close genetic relationship with the remaining others to reduce the bias in genetic diversity estimation.
Reproducibility	The data set was public available, and all analytical details were described in Methods. Analyses were run multiple times to make the results reproducible.
Randomization	Randomized experiments were not part of this study.
Blinding	Blind experiments were not part of this study.

Did the study involve field work? ☒ Yes ☐ No

Field work, collection and transport

Field conditions	Arid desert with limitation of transportation and electricity.
Location	Great Gobi National Park, part of Great Gobi-Strictly Protected Area "A" in Mongolia.
Access and import/export	The wild Bactrian camel samples were collected according to an agreement between Great Gobi National Park Administration of Mongolia and Inner Mongolia Agricultural University on May 08, 2012.
Disturbance	Local anesthesia and proper surgical procedures were adopted in the collection.

Reporting for specific materials, systems and methods

We require information from authors about some types of materials, experimental systems and methods used in many studies. Here, indicate whether each material, system or method listed is relevant to your study. If you are not sure if a list item applies to your research, read the appropriate section before selecting a response.

Materials & experimental systems

n/a	Involved in the study	
☒	☐	Antibodies
☒	☐	Eukaryotic cell lines
☒	☐	Palaeontology
☐	☒	Animals and other organisms
☒	☐	Human research participants
☒	☐	Clinical data

Methods

n/a	Involved in the study	
☒	☐	ChIP-seq
☒	☐	Flow cytometry
☒	☐	MRI-based neuroimaging

Animals and other organisms

Policy information about studies involving animals; ARRIVE guidelines recommended for reporting animal research

Laboratory animals	This study did not use laboratory animals.
Wild animals	The 19 wild Bactrian camels captured for DNA collection were part of the captive breeding herd in the protected area. Local anesthesia was applied to the ear, and the wound was disinfected with iodophor and sulfonamide powder.
Field-collected samples	The tissues collected were eluted with PBS buffered solutions, placed in cryotubes, and then transported with dry ice to the lab and stored at -80°C.
Ethics oversight	The collection of domestic camels was made during routine veterinary treatments with the guidelines from the Camel Protection Association of Inner Mongolia. The collection of wild camels was approved by the Great Gobi National Park.

Note that full information on the approval of the study protocol must also be provided in the manuscript.
